# Prevalence and predictors of low back pain among the Iranian population: Results from the Persian cohort study

**DOI:** 10.1016/j.amsu.2022.103243

**Published:** 2022-01-10

**Authors:** Mohammad Ghafouri, Azin Teymourzadeh, Amin Nakhostin-Ansari, Sadaf G. Sepanlou, Sahar Dalvand, Farhad Moradpour, Amir Hooshang Bavarsad, Shahrokh Sadeghi Boogar, Morteza Dehghan, Alireza Ostadrahimi, Javad Aghazadeh-Attari, Mahmood Kahnooji, Ali Hosseinipour, Ali Gohari, Seyed Vahid Hosseini, Masoud Mirzaei, Alireza Khorram, Mehdi Shahmoradi, Farhad Pourfarzi, Mahmood Moosazadeh, Fariborz Mansour-Ghanaei, Hossein Marioryad, Farid Najafi, Stephane Genevay, Navid Moghadam, Ramin Kordi

**Affiliations:** a Sports Medicine Research Center, Neuroscience Institute, Tehran University of Medical Sciences, Tehran, Iran; b Imam Khomeini Hospital Complex, Tehran University of Medical Sciences, Tehran, Iran; c Research Center for War-affected People, Tehran University of Medical Sciences, Tehran, Iran; d Digestive Disease Research Center, Digestive Diseases Research Institute, Tehran University of Medical Sciences, Tehran, Iran; e Department of Epidemiology and Biostatistics, School of Public Health, Tehran University of Medical Sciences, Tehran, Iran; f Liver and Pancreatobiliary Diseases Research Center, Digestive Diseases Research Institute, Tehran University of Medical Sciences, Tehran, Iran; g Social Determinants of Health Research Center, Research Institute for Health Development, Kurdistan University of Medical Sciences, Sanandaj, Iran; h Department of Medicine, Ahvaz Jundishapur University of Medical Sciences, Ahvaz, Iran; i Gastroenterohepatology Research Center, Shiraz University of Medical Sciences, Shiraz, Iran; j Department of Orthopedics, School of Medicine, And Modeling in Health Research Center, Shahrekord University of Medical Sciences, Shahrekord, Iran; k Nutrition Research Center, Tabriz University of Medical Sciences, Tabriz, Iran; l Social Determinants of Health Research Center, Urmia University of Medical Sciences, Urmia, Iran; m Department of Internal Medicine, Faculty of Medicine, Rafsanjan University of Medical Sciences, Rafsanjan, Iran; n Noncommunicable Diseases Research Center, Fasa University of Medical Sciences, Fasa, Iran; o Department of Clinical Biochemistry and Nutrition, Faculty of Medicine, Sabzevar University of Medical Sciences, Sabzevar, Iran; p Colorectal Research Center, Shiraz University of Medical Sciences, Shiraz, Iran; q Yazd Cardiovascular Research Centre, Shahid Sadoughi University of Medical Sciences, Yazd, Iran; r Health Promotion Research Center, Zahedan University of Medical Sciences, Zahedan, Iran; s Endocrinology and Metabolism Research Center, Hormozgan University of Medical Sciences, Bandar Abbas, Iran; t Digestive Disease Research Center, Ardabil University of Medical Sciences, Ardabil, Iran; u Gastrointestinal Cancer Research Center, Non-communicable Diseases Institute, Mazandaran University of Medical Sciences, Sari, Iran; v Gastrointestinal and Liver Diseases Research Center, Guilan University of Medical Sciences, Rasht, Iran; w Yasuj University of Medical Sciences, Yasuj, Iran; x Research Center for Environmental Determinants of Health (RCEDH), Health Institute, Kermanshah University of Medical Sciences, Kermanshah, Iran; y Division of Rheumatology, Faculty of Medicine, Geneva University Hospitals, Geneva, Switzerland

**Keywords:** LBP, low back pain, BMI, body mass index, YLD, years of healthy life lost due to disability, Prevalence, Low back pain, Iran, PERSIAN

## Abstract

**Background and objectives:**

Low back pain (LBP) is a common health condition in populations. Limited large-scale population-based studies evaluated the prevalence and predictors of LBP in developing countries. This study aimed to evaluate the prevalence and factors associated with LBP among the Iranian population.

**Methods:**

We used baseline information from the Prospective Epidemiological Research Studies in Iran (PERSIAN), including individuals from 16 provinces of Iran. LBP was defined as the history of back pain interfering with daily activities for more than one week during an individual's lifetime. Various factors hypothesized to affect LBP, such as age, sex, marital status, educational status, ethnicity, living area, employment status, history of smoking, body mass index (BMI), physical activity, sleep duration, wealth score, history of joint pain, and history of morning stiffness in the joints were evaluated.

**Results:**

In total, 163770 Iranians with a mean age of 49.37 (SD = 9.15) were included in this study, 44.8% of whom were male. The prevalence of LBP was 25.2% among participants. After adjusting for confounders, the female gender [OR:1.244(1.02–1.50)], middle and older ages [OR:1.23(1.10–1.33) and OR:1.13(1.07–1.42), respectively], being overweight or obese [OR:1.13(1.07–1.19) and OR:1.21(1.16–1.27), respectively], former and current smokers (OR:1.25(1.16–1.36) and OR:1.28(1.17–1.39), respectively], low physical activity [OR:1.07(1.01–1.14)], and short sleep duration [OR: 1.09(1.02–1.17)] were significantly associated with LBP.

**Conclusion:**

In this large-scale study, we found the lifetime prevalence of LBP to be lower among the Iranian population in comparison to the global prevalence of LBP; further studies are warranted to evaluate the causality of risk factors on LBP.

## Highlights

• The lifetime prevalence of low back pain in the Iranian population is 25.2%.
• Our survey includes 163770 people from 16 Iranian provinces.
• We used PERSIAN Cohort data which is the largest study conducted in Iran population.
• For analysis, we used both unadjusted and adjusted logistic regression models.

## 1 Introduction

Low back pain (LBP) is a common symptom that affects people of all ages [1]. The global point prevalence of LBP is about 12%, with a one-year prevalence of 38% and a lifetime prevalence of around 40%. In both developed and developing countries, LBP is the leading cause of years of healthy life lost due to disability (YLD), and it ranks sixth in terms of overall disease burden [2].

Over the next few decades, as the world's population ages, the number of people suffering from LBP is expected to rise dramatically [3]. In developed countries, the burden of LBP is expected to rise even further. Between 1990 and 2015, the YLD caused by LBP increased by 54% globally, with the greatest growth occurring in low- and middle-income countries [4].

LBP has several recognized risk factors. While certain risk factors of LBP, such as high body mass index (BMI) and occupational risks, have been well studied [5,6], the role of other contributing factors has not been well evaluated. Therefore, the identification and evaluation of risk factors will aid in the development of preventative measures and the reduction of LBP burden [7]. Furthermore, most researches on the epidemiology, clinical presentation, burden, psychosocial effect, and management of LBP have been performed in developed countries; as a result, little is known about these issues in developing and low-to middle-income countries [8].

Several studies have been conducted in Iran on the prevalence and risk factors of LBP in certain groups, including children, pregnant females, workers, and healthcare workers, which found a point prevalence of 15%–57% across different groups [[9], [10], [11], [12], [13], [14]]. However, their results may not be generalizable to the Iranian general population and there is still a need for more comprehensive studies on the prevalence and determinants of LBP in Iran. Therefore, this study aimed to evaluate the prevalence of LBP and its predictors among the Iranian population based on data from the Prospective Epidemiological Research Studies in Iran (PERSIAN Cohort).

## 2 Materials and methods

### 2.1 Design

A cross-sectional study was designed using data obtained from the PERSIAN Cohort Study. Since 2014, the PERSIAN cohort has enrolled about 165,000 Iranians between the ages of 35 and 70 from 18 cohort sites, to determine the burden of non-communicable diseases (NCDs) and examine the associated risk factors in Iran's various ethnic groups and geographical areas. The rationale and methodology of the PERSIAN Cohort study have been previously explained [15]. In brief, participants were enrolled at the various cohort sites, where biological samples were obtained and they were interviewed by qualified interviewers on various exposures. All data has been obtained using the same protocols and measurement tools. Participants are planned to be followed up for 15 years past enrollment, with the incidence of death and major NCDs being the main outcomes of the cohort study. For the purpose of the current study, data obtained at the enrollment phase of the PERSIAN Cohort has been used. After excluding individuals with missing data required for this study, a total of 163770 adults from 18 cohort sites in 16 different provinces of Iran were included in this analysis. This study has been reported in line with the STROCSS criteria [16] and also approved by the Tehran University of Medical Sciences' ethics committee, with the approval number IR.TUMS.NI.REC.1399.020. Also, the work has been reported in line with the STROCSS criteria [17].

### 2.2 Measurements

Back pain was described as a binary variable, with “yes” indicating that a respondent has experienced back pain that interfered with daily activities for more than one week during his/her life. Smoking status was defined as smoking at least 100 cigarettes during a lifetime based on the current National Health Interview Survey (NHIS) smoking definition [18]. According to the World Health Organization (WHO) classification, BMI was calculated using the formula BMI = weight (kg)/[height (m)]^2^. Individuals with BMI values of less than 18.5, 18.5–24.9, 25–29.9, and 30 kg/m2 or higher were categorized as underweight, normal, overweight, or obese, respectively [19]. Physical activity was evaluated using a validated questionnaire recording participants' self-reported daily activities over one year, using a compendium of physical activities and the metabolic equivalent of task (MET) score over 24 h was then calculated based on the activities reported [20,21]. We categorized physical activity levels to medium, lower, and higher than the average MET score in our analysis. Socioeconomic status was evaluated using a wealth score index (WSI), which was estimated by multiple correspondence analysis (MCA) of the variables listed ahead: access to a freezer, access to a washing machine, access to a dishwasher, access to a computer, internet access, access to a motorcycle, access to a car (no access, access to a car with price of <50 million Tomans, and access to a car with price of >50 million Tomans), access to a vacuum cleaner, color TV type (no color TV or regular color TV vs. Plasma color TV), owning a mobile, owning a personal computer (PC) or laptop, international trips during the lifetime (never, pilgrimage, both pilgrimage or non-pilgrimage trips). The lowest 20% of scores are given a quintile number of one, the next lowest 20% of scores are given a quintile number of two, and so on, up to the highest 20% of scores being given a quintile number of five. Because of the weighted analysis, there is a discrepancy in the percentages in each quintile [22]. Also, participants' sleep duration was evaluated based on participants’ self-reports of sleep duration during the night. We defined short, normal, and long sleep duration as less than 6 h of sleep, 6–8 h of sleep, and more than 8 h of sleep, respectively [23]. The joint pain and morning stiffness in joints were considered positive if the answer to questions “Having ever had joint pain “and “Having ever experienced joint pain with morning stiffness of 1 h or more were “yes”, respectively. Joint pain was referred to all joints other than the back.

### 2.3 Statistical analysis

We used sampling weights defined as the inverse probability of being selected in the survey based on the Iranian 2016 population and housing census data. To describe the data, we used descriptive statistics including frequency and percentages. The weighted prevalence of LBP and its 95% confidence intervals across categorical variables were reported. Chi-square test was used to examine the distribution of LBP across categories. We conducted univariable (unadjusted) and multivariable (adjusted) logistic regression models to assess the relationship between LBP and other variables under the study. All statistical analyses were performed by Stata software version 12 (Stata Corp, College Station, TX, USA).

## 3 Results

In this study, we enrolled 163770 people from Iran's 16 provinces. The mean age of participants was 49.37 (SD = 9.15), with 44.8% males and 55.2% females. Our participants came from both urban (70.8%) and rural (29.2%) areas. Table 1 summarizes the participants' characteristics in more detail.

**Table 1:** The baseline characteristics of the population under study.

Variable	Number (%)
Sex	
Male	73373 (44.8)
Female	90397 (55.2)
**Age (year)**	
35–44	58107 (35.5)
45–54	55435 (33.8)
55–64	39697 (24.2)
>65	10531 (6.4)
**BMI**	
Underweight	2974 (1.8)
Normal	42193 (25.9)
Overweight	66428 (40.8)
Obese	51299 (31.5)
**Ethnicity**	
Fars	49095 (30.0)
Azari	41450 (25.3)
Balouch	2968 (1.8)
Kurd	15317 (9.4)
Lor	8559 (5.2)
Arab	10336 (6.3)
Zaboli	5085 (3.1)
Gilak	9443 (5.8)
Turk nomad	4849 (3.0)
Arab nomad	3401 (2.1)
Mazani	9149 (5.6)
Mixed	4118 (2.5)
**Living area**	
Urban	115979 (70.8)
Rural	47791 (29.2)
**Education years**	
Illiterate	33675 (20.6)
Primary school	51974 (31.8)
Guidance school	23132 (14.1)
High school	35223 (21.5)
University	19529 (11.9)
**Employment status**	
Employed	72973 (44.6)
Unemployed	22190 (13.5)
Housewife	60079 (36.7)
Retired	8528 (5.2)
**Smoking status**	
Smoker	22951 (14.1)
Non smoker	127490 (78.2)
former	12528 (7.7)
**Socioeconomic Status**	
1st quintile	32643 (20.0)
2nd quintile	34667 (21.3)
3rd quintile	33536 (20.6)
4th quintile	35486 (21.8)
5th quintile	26731 (16.4)
**Marital Status**	
Single	14648 (8.9)
Married	149122 (91.1)
**Physical activity (MET)**	
Lower than average	54624 (33.4)
Medium	54556 (33.3)
Higher than average	54590 (33.3)

Overall, 41045 participants had experienced LBP, yielding a lifetime prevalence of 25.2%. The prevalence of LBP was significantly higher in females in comparison to males [30.3% (23.7–37.9) vs. 21.2% (16.7–26.7), respectively (p < 0.0001)]. LBP was also significantly more prevalent in older ages, obese individuals, those from the Mazani ethnicity, individuals living in rural areas, those who were illiterate, housewives, participants with no history of smoking, those exerting higher physical activity, singles, and individuals with short sleep durations (Table 2).

**Table 2:** **Weighted** Prevalence of low back pain in different basic and demographic groups.

Variable	Prevalence of low back pain, number (%)	95% Confidence Interval
**Sex**^b^		
Male	14795 (21.2)	16.7–26.7
Female	26250 (30.3)	23.7–37.9
**Age (year)**^b^		
35–44	12302 (21.4)	16.8–26.8
45–54	14756 (27.8)	22.2–34.2
55–64	11099 (30.4)	23.9–37.8
>65	2888 (30.8)	23.4–39.4
**BMI (Kg/m2)**^b^		
Underweight	495 (15.1)	11.6–19.4
Normal	8750 (21.7)	16.3–28.1
Overweight	16498 (25.5)	20.3–31.5
Obese	15154 (30.2)	24.4–36.7
**Ethnicity**^b^		
Fars	10855 (22.5)	17.2–28.8
Azari	10040 (25.4)	21.6–29.5
Balouch	607 (18.8)	16.8–20.9
Kurd	4275 (28.0)	22.7–34.0
Lor	1323 (16.5)	15.3–17.9
Arab	3116 (30.2)	26.3–34.2
Zaboli	1068 (18.5)	18.5–18.6
Gilak	2652 (27.9)	27.0–28.8
Turk nomad	914 (18.4)	17.1–19.8
Arab nomad	1047 (25.2)	14.3–40.4
Mazani	4115 (42.0)	41.9–42.1
Mixed	1033 (25.2)	19.0–32.7
**Living area**^b^		
Urban	29255 (24.9)	19.9–30.5
Rural	11790 (28.6)	21.1–37.5
**Education**^b^		
Illiterate	10180 (32.5)	25.2–40.8
Primary school	13455 (27.3)	22.0–33.4
Guidance school	5306 (23.9)	18.2–30.7
High school	7990 (23.5)	18.4–29.4
University	4106 (22.0)	16.2–29.1
**Employment status**^b^		
Employed	15053 (21.4)	16.5–27.3
Unemployed	6195 (27.4)	20.8–35.1
Housewife	17678 (31.5)	23.8–40.4
Retired	2119 (26.5)	21.1–32.8
**Smoking history**^b^		
Smoker	5173 (23.4)	19.1–28.4
Non smoker	32813 (26.2)	20.4–33.0
former	3041 (24.9)	19.4–31.3
**Physical activity (MET)**^a^		
low	14440 (25.6)	20.9–31.0
medium	13739 (25.4)	20.3–31.3
high	12866 (26.0)	19.5–33.8
**Socioeconomic Status**^b^		
1st quintile	8698 (28.3)	19.8–38.8
2nd quintile	8635 (25.8)	20.2–32.3
3rd quintile	8192 (24.8)	20.4–29.8
4th quintile	8989 (25.9)	20.8–31.9
5th quintile	6447 (24.4)	19.2–30.6
**Marital status**^b^		
Single	4022 (28.5)	22.6–35.3
Married	37023 (25.5)	20.1–31.8
**Morning stiffness in joint**^b^		
Positive	5574 (54.0)	43.3–64.3
Negative	35470 (24.2)	18.9–30.3
**Joint pain**^b^		
Positive	27968 (35.5)	28.3–43.4
Negative	13077 (16.1)	11.9–21.5
**Sleep duration**^b^		
Short	6251 (27.5)	20.5–35.8
Normal	25725 (25.0)	19.6–31.3
Long	8970 (26.8)	22.0–32.1

a P-value < 0.05.

b P-value < 0.0001.

Results of the logistic regressions are shown in Table 3. After adjusting for other variables, we found that the female gender, age category of 55–64, higher BMI, some ethnicities, smoking, low physical activity, short sleep duration, 4th quintile socioeconomic status, morning stiffness in joints, and joint pain are significantly associated with LBP (P < 0.05), while employment status, education, and marital status were no longer significantly correlated with LBP.

**Table 3:** Univariable (unadjusted) and multivariable (adjusted) logistic regression analysis for Low Back Pain and its Related Factors.

Variable	Unadjusted OR (95% CI)	p-value	Adjusted OR (95% CI)	p-value
**Sex**				
Male	Reference		Reference	
Female	1.612 (1.347–1.930)	<0.0001	1.244 (1.028–1.504)	0.027
**Age (Year)**				
35–44	Reference		Reference	
45–54	1.415 (1.307–1.513)	<0.0001	1.214 (1.103–1.336)	0.001
55–64	1.608 (1.432–1.805)	<0.0001	1.237 (1.074–1.423)	0.005
>65	1.636 (1.352–1.980)	<0.0001	1.131 (0.895–1.430)	0.283
**BMI (Kg/m2)**				
Normal	Reference		Reference	
Underweight	0.644 (0.546–0.761)	<0.0001	0.773 (0.693–0.863)	<0.0001
Overweight	1.236 (1.160–1.317)	<0.0001	1.135 (1.079–1.195)	<0.0001
Obese	1.564 (1.431–1.708)	<0.0001	1.218 (1.160–1.279)	<0.0001
**Ethnicity**				
Fars	Reference		Reference	
Azari	1.172 (0.796–1.172)	0.398	1.167 (0.775–1.757)	0.438
Balouch	0.796 (0.574–0.796)	0.157	0.858 (0.616–1.195)	0.343
Kurd	1.341 (0.871–1.341)	0.169	1.374 (0.944–2.001)	0.092
Lor	0.682 (0.501–0.682)	0.018	0.680 (0.525–0.881)	0.006
Arab	1.492 (1.206–1.492)	0.001	1.500 (1.187–1.895)	0.002
Zaboli	0.784 (0.563–0.784)	0.138	0.862 (0.638–1.164)	0.311
Gilak	1.330 (0.950–1.330)	0.091	1.271 (0.945–1.710)	0.106
Turk nomad	0.776 (0.603–0.776)	0.050	0.817 (0.577–1.155)	0.235
Arab nomad	1.159 (0.584–1.159)	0.656	1.194 (0.530–2.688)	0.651
Mazani	2.494 (1.790–2.494)	<0.0001	2.808 (2.101–3.753)	0.000
Mixed	1.162 (0.804–1.162)	0.401	1.169 (0.827–1.653)	0.353
**Living area**				
Urban	Reference		Reference	
Rural	1.208 (0.941–1.551)	0.128	1.034 (0.847–1.263)	0.727
**Education**				
Illiterate	Reference		Reference	
Primary school	0.780 (0.679–0.859)	0.001	0.943 (0.869–1.024)	0.154
Guidance school	0.652 (0.558–0.762)	<0.0001	0.930 (0.822–1.053)	0.236
High school	0.636 (0.531–0.762)	<0.0001	0.927 (0.800–1.074)	0.291
University	0.585 (0.458–0.747)	<0.0001	0.882 (0.722–1.078)	0.204
**Employment status**				
Employed	Reference		Reference	
Unemployed	1.383 (0.903–2.121)	0.127	1.130 (0.920–1.390)	0.227
Housewife	1.687 (1.401–2.031)	<0.0001	1.151 (0.938–1.413)	0.165
Retired	1.326 (1.162–1.514)	<0.0001	1.126 (0.961–1.319)	0.134
**Smoking history**				
Non-smoker	Reference		Reference	
Former smoker	0.932 (0.793–1.096)	0.373	1.259 (1.164–1.361)	<0.0001
Current smoker	0.861 (0.714–1.038)	0.110	1.28 (1.178–1.392)	<0.0001
**Physical activity**				
Medium	Reference		Reference	
Low	1.012 (0.922–1.110)	0.789	1.078 (1.015–1.145)	0.018
High	1.035 (0.891–1.202)	0.637	1.032 (0.986–1.080)	0.168
**Marital Status**				
Single	Reference		Reference	
Married	0.856 (0.763–0.960)	0.011	1.044 (0.964–1.129)	0.272
**Sleep duration**				
Normal	Reference		Reference	
Short	1.137 (1.016–1.271)	0.028	1.095 (1.02–1.175)	0.015
Long	1.096 (0.998–1.203)	0.055	1.038 (0.985–1.094)	0.155
**Socioeconomic status**				
1st quintile	Reference		Reference	
2nd quintile	0.879 (0.725–1.068)	0.181	0.993 (0.874–1.127)	0.903
3rd quintile	0.836 (0.615–1.135)	0.233	1.075 (0.916–1.26)	0.354
4th quintile	0.888 (0.688–1.146)	0.339	1.17 (1.017–1.346)	0.030
5th quintile	0.819 (0.625–1.072)	0.136	1.18 (1.01–1.378)	0.038
**Morning stiffness in joint**				
Negative	Reference		Reference	
Positive	3.681 (2.568–5.276)	<0.0001	2.128 (1.544–2.933)	<0.0001
**Joint pain**				
Negative	Reference		Reference	
Positive	2.863 (2.382–3.442)	<0.0001	2.538 (2.231–2.887)	<0.0001

## 4 Discussion

In this study, we evaluated the lifetime prevalence of LBP in a large sample of Iranians, which to the best of our knowledge, is the largest study in the Middle East region. We also evaluated the potential factors that are associated with LBP. We found the prevalence of LBP to be 25.2% in the Iranian population, which is considerably lower than estimates reported in other populations. Recent studies have revealed a lifetime prevalence of 75.8% in Serbia [24], 47% in African countries [25], and 65%–80% in the United States (US) [26]. Hoy et al. found that the global lifetime prevalence of LBP is about 38.9%.

Two previous studies conducted in Iran in 2012 and 2017, reported a 29.3% and 27% prevalence of LBP, respectively [7,27]. Several methodological factors are different among our study and these previous studies, yielding different results [28]. First, the definition of having LBP varied, with previous studies evaluating LBP in the few months preceding study conduction, while in our study, the lifetime prevalence was considered. At the same time, our results may be affected by memory lapses while in the other studies, it is more likely that LBP is reported without recall bias. In addition, given the longitudinal nature of cohort studies, most of the PERSIAN Cohort sites included small cities with small migration rates to limit loss to follow-up, while the previous studies included large, metropolitan cities such as Tehran, Shiraz, and Isfahan, in which the prevalence of LBP may be different due to lifestyle factors. Our sample size, on the other hand, was considerably larger [163770 vs. 25307 (2012) and 78898 (2017)], and includes individuals from various types of lifestyles and occupations, from office employees to drivers, farmers, ranchers, etc. that are not represented by estimates obtained from larger cities [7,27].

After adjusting for other variables, we discovered that those of Mazani ethnicity have the strongest significant correlation with LBP. It may be owing to their intense work, as many of them work in the rice agriculture.

We found that the prevalence of LBP is higher in females than males (30.3% versus 21.2%), which is consistent with the findings of other studies [29,30]. Females are biologically susceptible to developing LBP due to risk factors such as pregnancy, contraceptive use, and estrogen use during menopause [31,32]. Furthermore, postmenopausal and older females have an increased risk of LBP than their opposite-sex counterparts [33,34]. Housewives also have a significantly higher prevalence of LBP than people with occupations, as we found in our study. In total, a higher prevalence of LBP among females and a higher risk of developing LBP in this group indicate that Iranian females may be one of the groups that benefit from interventions for primary prevention of LBP.

Prevalence of LBP was also significantly higher in individuals with a higher BMI, with the strongest association being seen in the overweight and obese categories. While several theories have been proposed to explain the association between obesity and LBP, loss of muscle mass in the trunk and lower extremities could be the main cause [35]. Differences in the distribution of body fat mass or the proportion of lean body mass may also be the reason [36,37]. Given the significant prevalence of obesity in our study population (31.5%), efforts to reduce obesity may have extra benefits in terms of affecting LBP in the Iranian community and can be considered as a potential intervention for reducing the prevalence of LBP in the Iranian population [38].

PERSIAN Cohort participants with lower socioeconomic status and educational attainment had a higher prevalence of LBP. Various studies have shown the positive impact of higher income, better socioeconomic status, wealth, and education on LBP. Higher sensitivity to pain, poor access to healthcare, and a lack of knowledge about how to seek proper help in time are the reasons that lead to a higher prevalence of LBP in the opposite groups [[39], [40], [41]]. Another probable theory of lower prevalence of LBP among individuals with high socioeconomic status, high educational status, and employed people is due to their healthy behaviors and more social support in these groups [[41], [42], [43], [44]]. Indeed, health literacy is likely to be more important than an educational degree, and different generations may be educated differently about health [45]. Improving health literacy among people with lower socioeconomic status may positively impact LBP in people with lower socioeconomic status.

Our findings suggest that we can consider healthy sleep time as a protective factor for LBP. Some studies have shown how sleep disturbances can lower the pain threshold and cause more intense LBP [46,47]. It is discussed that people with sleep problems seem to have a higher risk of developing conditions such as depression, anxiety, fibromyalgia, and migraine, which lower mental ability to cope with pain [48,49]. Thus, there is likely a bidirectional relationship between sleep and LBP that needs to be substantiated by further studies [50].

### 4.1 Strengths and limitations

This was the largest survey assessing LBP in the Middle East population to the best of our knowledge. In addition, the study design included people of different ethnicities, socioeconomic statuses, living areas, and the high diversity of participants increases the generalizability of our findings. In addition, the number of factors assessed in the study made the results more valuable and accurate after adjusting for other risk factors.

This study has several limitations as well. First, the data on symptoms and their duration is based on self-reports, and recall bias and memory lapses could affect the data accuracy. Also, while many possible contributing factors have been reported, the cross-sectional design is unable to determine causality between them and LBP. In addition, this study was part of the PERSIAN Cohort Study, which aimed to assess the health status of the Iranian population more comprehensively and does not specialize in the assessment of LBP and its risk factors. As a result, we lacked additional details such as the various stages of LBP and the duration and intensity of LBP in individuals.

## 5 Conclusion

In this large-scale study, we found that lifetime LBP is lower among the Iranian population than the other countries and the global prevalence of LBP. Also, we found that female gender, higher BMI, smoking, low physical activity, short sleep duration, and history of other musculoskeletal symptoms were significantly correlated with LBP after adjusting for other variables.

## Ethical approval

Our study used PERSIAN cohort data which its design is published elsewhere. The design of the PERSIAN Cohort Study was approved by the ethics committees of the Ministry of Health and Medical Education, the Digestive Diseases Research Institute (Tehran University of Medical Sciences), and each participating university. Also, the current study was approved by the Tehran University of Medical Sciences' ethics committee, with the approval number IR.TUMS.NI.REC.1399.020.

## Please state any sources of funding for your research

The research had no sponsor or funding source.

## Author contribution

Conceptualization, Navid Moghadam, Ramin Kordi, All others, Data Curation, Farhad Moradpour, Amir Hooshang Bavarsad, Shahrokh Sadeghi Boogar, Morteza Dehghan, Alireza Ostadrahimi, Javad Aghazadeh-Attari, Mahmood Kahnooji, Ali Hosseinipour, Ali Gohari, Seyed Vahid Hosseini, Masoud Mirzaei, Alireza Khorram, Mehdi Shahmoradi, Farhad Pourfarzi, Mahmood Moosazadeh, Fariborz Mansour-Ghanaei, Hossein Marioryad, Farid Najafi, Formal Analysis, Sadaf G. Sepanlou, Investigation, Mohammad Ghafouri, Methodology, Sadaf G. Sepanlou, Sahar Dalvand, Project Administration, Mohammad Ghafouri, Navid Moghadam, Software, Sadaf G. Sepanlou, Supervision, Mohammad Ghafouri, Navid Moghadam, Ramin Kordi, Writing – Original Draft Preparation, Mohammad Ghafouri, Azin Teymourzadeh, Writing – Review & Editing, Azin Teymourzadeh, Amin Nakhostin-Ansari, Stephane Genevay, All others.

## Please state any conflicts of interest

The authors have no conflicts of interest to declare.

## Consent

Prior to participating in the study, participants provide written approval in the form of a consent form.

## Registration of research studies

researchregistry7464.

## Guarantor

Navid Moghadam.

## Provenance and peer review

Not commissioned, externally peer reviewed.
